# Malignant *MITF*‐Rearranged PEComa With Novel *EEF1A1::MITF* Fusion

**DOI:** 10.1002/gcc.70168

**Published:** 2026-08-31

**Authors:** Sierra Raney, Arivarasan Karunamurthy, Jonhan Ho, Robert Bubar, Maedeh Mohebnasab, John Skaugen, Mohamed Elmoryah, Benjamin Nacev, Richard McGough, Ivy John

**Affiliations:** ^1^ Department of Pathology University of Pittsburgh Pittsburgh Pennsylvania USA; ^2^ Department of Medicine University of Pittsburgh Pittsburgh Pennsylvania USA; ^3^ UPMC Hillman Cancer Center Pittsburgh Pennsylvania USA; ^4^ Department of Orthopaedic Surgery University of Pittsburgh Pennsylvania USA

**Keywords:** *EEF1A1::MITF*, *MITF*, MITF‐overexpressing PEComa, PEComa

## Abstract

*MITF*‐rearranged PEComas are rare, and their relationship to *MITF*‐altered melanocytic tumors remains incompletely characterized. We report a malignant superficial soft tissue neoplasm of the thigh in a 45‐year‐old woman harboring a novel *EEF1A1::MITF* fusion. The tumor was composed of epithelioid to plump spindle cells arranged in sheets, nests, and short fascicles with prominent capillary vasculature. Significant cytologic atypia, brisk mitotic activity, and necrosis were present. The tumor cells showed diffuse strong nuclear MITF and diffuse GPNMB expression, with patchy SMA, scattered desmin, and multifocal cathepsin K positivity, while S100, SOX10, HMB45, Melan A, PRAME, tyrosinase, pancytokeratin, CD34, and TFE3 were negative. Although atypical for classic PEComa, this immunophenotype resembles that described in MITF‐overexpressing PEComas. Molecular studies identified an *ATRX* frameshift mutation, homozygous *CDKN2A/CDKN2B* deletion, complex copy number alterations, and an in‐frame *EEF1A1::MITF* fusion. No canonical *TSC1*, *TSC2*, or *FLCN* alteration was identified. These findings expand the molecular spectrum of PEComa and support the emerging concept that a subset of cutaneous PEComas may be driven by *MITF* rearrangements.

## 1 Introduction

PEComas are uncommon mesenchymal neoplasms with variable smooth muscle and melanocytic differentiation. Most are associated with mTOR pathway dysregulation, most commonly through inactivating alterations in *TSC1* or *TSC2*, whereas a smaller subset harbors *TFE3* gene fusions [1, 2]. More recently, PEComas with diffuse MITF expression, including rare tumors with *MITF* rearrangement, have been described [3]. The recognition of *MITF*‐altered melanocytic tumors, including those with *ACTIN::MITF* and *MITF::CREM* fusions, has blurred the boundary between these tumors and PEComa [4, 5]. We report a malignant superficial soft tissue neoplasm with a novel *EEF1A1::MITF* fusion, expanding the spectrum of *MITF*‐rearranged PEComa.

## 2 Case Presentation

A 45‐year‐old woman presented with a right thigh mass of 1 month duration. Ultrasonography showed a superficial heterogeneous irregular lesion measuring 2.6 cm in greatest dimension without vascular flow. After developing tenderness, erythema, and warmth, she underwent excisional biopsy of the lesion. Computed tomography (CT) performed after biopsy demonstrated a heterogeneous infiltrative mass within the lateral subcutaneous tissues measuring 4.6 × 4.4 × 4.9 cm, extending into the overlying skin but separated from the deep fascia by a preserved fat plane. CT of the chest and abdomen showed no evidence of metastatic disease, and the patient underwent resection with negative margins followed by adjuvant radiation therapy. MRI performed 3 months later showed enhancing subcutaneous nodules in the lateral thigh and nodular enhancement along the resection margins, extending into the lateral aspect of the anterior compartment with vastus lateralis infiltration and deep into the posterior compartment. Biopsy confirmed recurrent disease.

**FIGURE 1:**
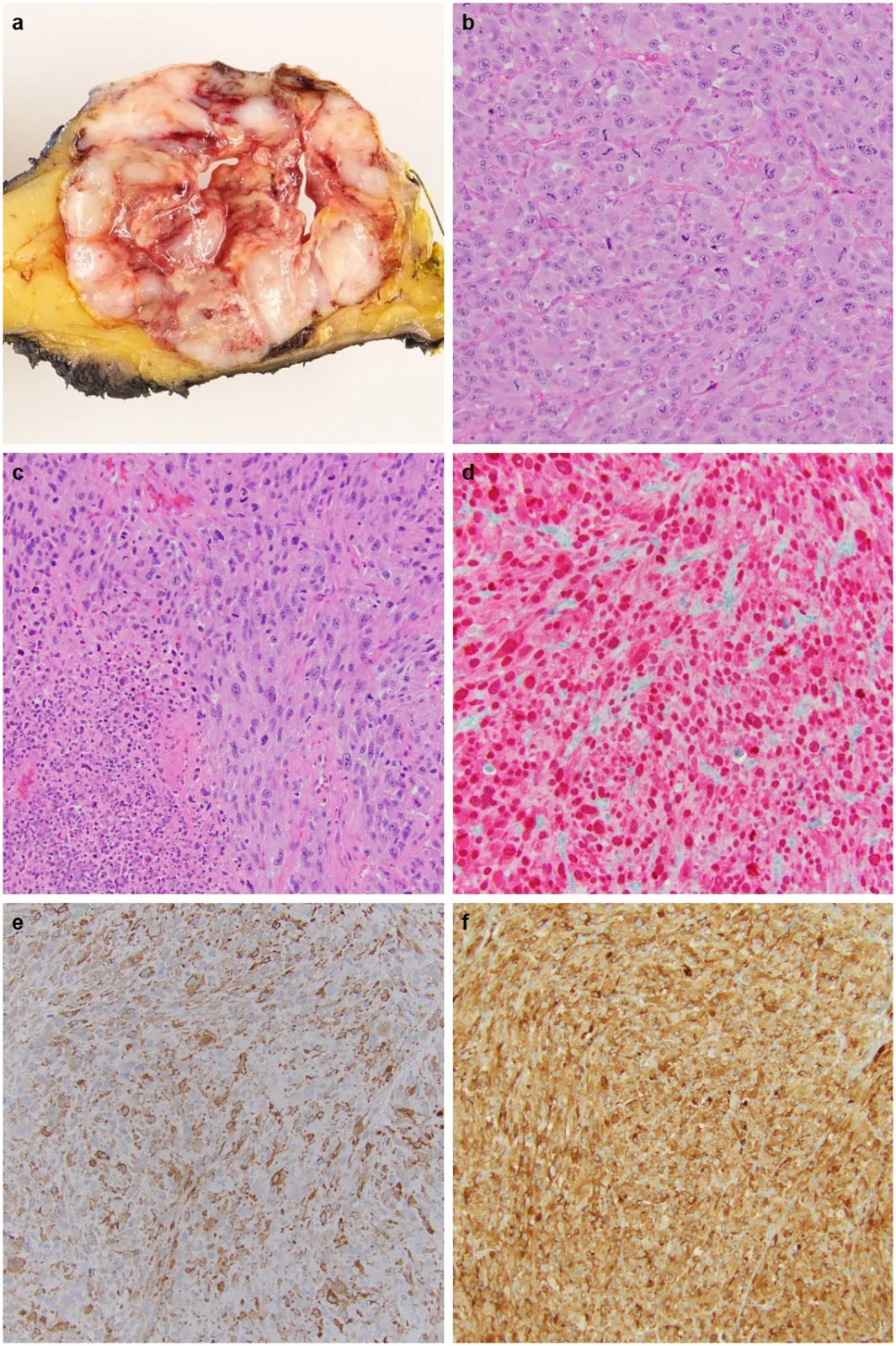
Gross examination showed a 6.2‐cm lobulated tan‐white mass with central necrosis involving the dermis and subcutis (A). Histologic sections demonstrated a nested growth pattern with a prominent vascular architecture (B). In other areas, the tumor cells assumed a more spindled morphology and were associated with necrosis (C). Immunohistochemically, the tumor cells showed diffuse strong nuclear MITF expression (D), patchy SMA positivity (E), and diffuse strong GPNMB expression (F).

**FIGURE 2:**
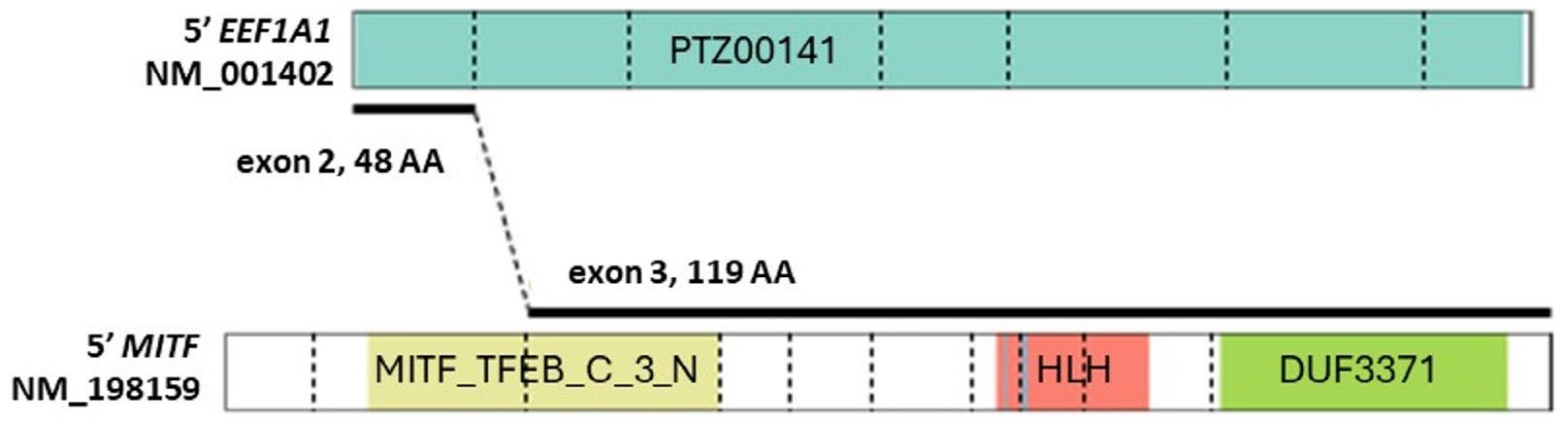
Model schematic of the resulting *EEF1A1::MITF* fusion protein product (created using https://pecan.stjude.org/proteinpaint). Whole transcriptome sequencing (RNA‐seq) identified a fusion involving exon 2 of *EEF1A1*and exon 3 of *MITF*, resulting in a predicted in‐frame fusion product. The preserved helix–loop–helix leucine zipper domain (HLH) of *MITF* is denoted in red.

Gross examination revealed a well‐defined, 6.2 cm lobulated mass with a partially necrotic tan‐white cut surface and focal areas of hemorrhage (Figure 1a). Histologic examination of the excisional biopsy and resection specimen showed an epithelioid to plump spindle cell neoplasm with abundant pale eosinophilic cytoplasm arranged in sheets, short fascicles, and nests, often surrounded by thin‐walled capillary vessels (Figure 1b). A subset of tumor cells showed significant cytologic atypia and pleomorphism, including enlarged vesicular nuclei with conspicuous nucleoli. Significant mitotic activity (> 50 per 10 high‐power fields) and multifocal areas of necrosis were noted (Figure 1c). The tumor cells showed diffuse strong nuclear MITF expression and diffuse expression with GPNMB, with patchy SMA, scattered desmin, and multifocal cathepsin K positivity (Figure 1d–f). The tumor cells were negative for S100, SOX10, HMB45, Melan A, PRAME, tyrosinase, caldesmon, CD34, pancytokeratin, and TFE3. Targeted next‐generation sequencing identified an *ATRX* frameshift mutation (p.N260Tfs*28), homozygous deletion of *CDKN2A/CDKN2B*, and complex copy number alterations including losses of 1q, 3p, 8q, 10p, 10q, 13q, 14q, 16q, 17p, and Xp, with gains of 7p and 17q. The tumor was microsatellite stable with a tumor mutational burden of 4.7 mutations/Mb. RNA‐based fusion analysis identified an *EEF1A1::MITF* fusion (5′ breakpoint: chr6:74229606; 3′ breakpoint: chr3:69986973), resulting in a predicted in‐frame fusion product (Figure 2). DNA methylation profiling classified the lesion as undifferentiated sarcoma (score 0.99).

## 3 Discussion

The main diagnostic question is whether this tumor is best regarded as a PEComa or as part of the emerging group of *MITF*‐altered melanocytic tumors. The overall findings favor classification as a PEComa. Morphologically, the tumor showed nested and fascicular growth, abundant pale eosinophilic cytoplasm, and a prominent capillary vasculature, features supportive of PEComa. The tumor did not express S100, SOX10, HMB45, Melan A, PRAME, or tyrosinase, but demonstrated diffuse MITF and GPNMB expression with patchy SMA, scattered desmin, and multifocal cathepsin K positivity. Although unusual for classic PEComa, this immunophenotype resembles that described in MITF‐overexpressing PEComas, which may show limited or absent expression of conventional melanocytic markers [3].

The molecular findings also support classification as a malignant PEComa. In the recently reported series of MITF‐overexpressing PEComas, increased MITF expression was most often linked to increased *MITF* copy number, with supernumerary copies identified in 20 of 36 tumors [3]. A driver event involving *TSC1*, *TSC2*, or *FLCN* was detected in 11 of 15 sequenced tumors, consistent with established PEComa pathogenesis. Of note, two benign tumors in that cohort showed *MITF* rearrangement. One harbored an *ACTG1::MITF* fusion identical to that reported in a clear cell tumor with melanocytic differentiation, whereas RNA sequencing failed in the other case and the fusion partner could not be identified. No *TSC1*, *TSC2*, or *FLCN* alteration was reported in the *ACTG1::MITF* case, while the driver status of the second *MITF*‐rearranged case was not determined. Malignant tumors in the same series demonstrated complex chromosomal copy number profiles and additional alterations involving *TP53*, *RB1*, and *ATRX*. Although such secondary alterations are not specific to PEComa, the present tumor shows molecular features similar to those described in malignant PEComas, including complex copy‐number alterations, homozygous *CDKN2A/CDKN2B* deletion, and an *ATRX* frameshift mutation.

The novel molecular finding in this case is the *EEF1A1::MITF* fusion. To our knowledge, *EEF1A1* has not previously been reported as an *MITF* fusion partner in PEComas or melanocytic neoplasia. *EEF1A1* has been described as a fusion partner in other mesenchymal tumors, including *PLAG1*‐rearranged pediatric fibromyxoid soft tissue tumor and lipoblastoma [6, 7]. *EEF1A1* is a highly expressed housekeeping gene encoding eukaryotic translation elongation factor 1‐alpha 1. As the 5′ fusion partner, *EEF1A1* likely provides a strong constitutive promoter driving MITF overexpression, analogous to the proposed mechanism of *ACTB::MITF* and *ACTG1::MITF* fusions. The fusion detected in this case is predicted to be in‐frame and joins *EEF1A1* to *MITF* exon 3, preserving the MITF basic helix–loop–helix leucine zipper domain. The absence of *TSC1*, *TSC2*, or *FLCN* alterations in this case raises the possibility that *EEF1A1::MITF* may serve as the primary oncogenic driver through aberrant MITF overexpression. However, the therapeutic implications of this finding are uncertain.

This case also highlights the overlap between PEComa and the emerging group of *MITF*‐altered melanocytic tumors, such as those with *ACTIN::MITF* and *MITF::CREM* fusions. *MITF::CREM*‐rearranged tumors may show cytologic atypia and increased mitotic activity, but they typically show a more conventional melanocytic immunophenotype, including diffuse S100, SOX10, and MITF expression [5, 8, 9, 10]. In contrast, the present tumor lacked S100, SOX10, HMB45, Melan A, PRAME, and tyrosinase, and showed diffuse strong GPNMB expression, patchy SMA, scattered desmin, multifocal cathepsin K expression, and PEComa‐like nested architecture with a prominent capillary vascular pattern. Most reported *ACTIN::MITF*‐rearranged tumors have been relatively bland, dermal‐based lesions composed of cells with clear to eosinophilic cytoplasm, low mitotic activity, and limited evidence of aggressive behavior [4, 11]. Their immunophenotype is variable, with consistent MITF expression and frequent HMB45 positivity, whereas Melan‐A, tyrosinase, S100, and SOX10 are not uniformly expressed. One exception is the rapidly enlarging *ACTG1::MITF*‐rearranged tumor reported by Bigot et al., which showed rapid growth, ulceration, cytologic atypia, increased mitotic activity, diffuse MITF expression, and focal HMB45 and desmin expression [12]. Bigot et al. emphasized the morphologic and immunophenotypic overlap with PEComa and noted that the tumor would be classified as malignant under proposed PEComa criteria. More recently, Mansour et al. expanded the *ACTIN::MITF*‐rearranged tumor literature by reporting five additional cases and comparing them with previously reported primary cutaneous PEComas [11]. Their study highlighted striking clinicopathologic overlap between these groups, including frequent MITF overexpression in primary cutaneous PEComas, and suggested that *MITF* alterations may drive a subset of primary cutaneous PEComas. In their literature review, Mansour et al. noted that the molecular background of primary cutaneous PEComas remains incompletely characterized and that available data suggest they typically lack *TSC1/TSC2* or *TFE3* alterations, although comprehensive fusion testing has not been uniformly performed.

The methylation classifier result of undifferentiated sarcoma should be interpreted in the context of the integrated findings. Methylation profiling is a useful ancillary tool in the diagnosis of mesenchymal tumors and their mimics, but current classifiers are limited by the diversity and size of their reference cohorts. Thus, underrepresented rare or recently recognized tumors may be assigned to the nearest well‐represented methylation class [13].

In summary, we report a malignant superficial soft tissue PEComa with a novel *EEF1A1::MITF* fusion, *ATRX* mutation, homozygous *CDKN2A/CDKN2B* deletion, and complex copy‐number alterations, expanding the molecular spectrum of PEComa and supporting a role for *MITF* rearrangements in a subset of cutaneous PEComas. Considered together with the previously reported *ACTG1::MITF*‐fused tumor described by Bigot et al., which likely represents the first reported malignant PEComa with an *MITF* fusion, the present tumor represents the second such case.

## Ethics Statement

This study was approved by the Institutional Review Board.

## Data Availability

The data that support the findings of this study are available on request from the corresponding author. The data are not publicly available due to privacy or ethical restrictions.
